# High performance broadband photodetector using fabricated nanowires of bismuth selenide

**DOI:** 10.1038/srep19138

**Published:** 2016-01-11

**Authors:** Alka Sharma, Biplab Bhattacharyya, A. K. Srivastava, T. D. Senguttuvan, Sudhir Husale

**Affiliations:** 1 Academy of Scientific and Innovative Research (AcSIR), National Physical Laboratory, Council of Scientific and Industrial Research, Dr. K. S Krishnan Marg, New Delhi-110012, India; 2 National Physical Laboratory, Council of Scientific and Industrial Research, Dr. K. S Krishnan Marg, New Delhi-110012, India

## Abstract

Recently, very exciting optoelectronic properties of Topological insulators (TIs) such as strong light absorption, photocurrent sensitivity to the polarization of light, layer thickness and size dependent band gap tuning have been demonstrated experimentally. Strong interaction of light with TIs has been shown theoretically along with a proposal for a TIs based broad spectral photodetector having potential to perform at the same level as that of a graphene based photodetector. Here we demonstrate that focused ion beam (FIB) fabricated nanowires of TIs could be used as ultrasensitive visible-NIR nanowire photodetector based on TIs. We have observed efficient electron hole pair generation in the studied Bi_2_Se_3_ nanowire under the illumination of visible (532 nm) and IR light (1064 nm). The observed photo-responsivity of ~300 A/W is four orders of magnitude larger than the earlier reported results on this material. Even though the role of 2D surface states responsible for high reponsivity is unclear, the novel and simple micromechanical cleavage (exfoliation) technique for the deposition of Bi_2_Se_3_ flakes followed by nanowire fabrication using FIB milling enables the construction and designing of ultrasensitive broad spectral TIs based nanowire photodetector which can be exploited further as a promising material for optoelectronic devices.

TIs are considered as a new class of materials revealing new phases of quantum matter, possessing conducting surface states while showing bulk insulating properties and have triggered many fundamental investigations on quantum behaviours of exotic quasi-particles^1^,^2^. The fascinating potential applications of TIs based devices include quantum computing^1^, dissipationless electronics^3^, spintronics^4^,^5^, enhanced thermoelectric effects^6^, optical recording^7^, high performance field effect transistor^8^, near infrared flexible electrodes^9^, thermoelectric and infrared applications^10^, laser photonics and high speed optoelectronic devices^11^,^12^,^13^,^14^,^15^ etc. Recently, Bi_2_Se_3_ TI has been studied for its special electronic properties, namely, the formation of a single Dirac cone inside a large bulk band gap (0.35 eV) by the surface states or a single Dirac cone on the surface^1^,^16^ and it is demonstrated as a 3D TI material^17^. Further, the work done on observation of topological surface state using quantum hall effect has demonstrated that the conduction occurs due to surface states^18^.

Bi_2_Se_3_ shows tunable surface bandgap (can be tuned by layer thickness) which is a key requirement of many optoelectronic devices. Thickness and size dependent interesting and exciting light absorption properties of Bi_2_Se_3_ has been recently studied^12^,^19^. The UV-visible absorption spectra of liquid phase exfoliated few layers of Bi_2_Se_3_ has shown enhanced light absorption in the visible region compared to bulk sample^12^. A very important dynamic property of photocurrent generation in exfoliated Bi_2_Se_3_ nanodevices has shown that the polarization of light can control the generation of photocurrents^20^. Ultra-thin nanosheets of Bi_2_Se_3_ have been successfully employed for the photocurrent studies^21^. Thus nanostructures of TIs are excellent systems to investigate the photodetector based applications and already high performance broadband TIs based photodetector has been proposed^22^. Despite the reported exciting electro-optical properties, ultrahigh sensitive broad spectral photodetector properties of TIs employing Bi_2_Se_3_ nanowires fabricated from high quality exfoliated flakes are largely remained elusive and have not been studied yet.

Here we report the broadspectral photo response of Bi_2_Se_3_ nanowires fabricated by a focused ion beam microscope. Significant photocurrent is observed when nanowires are illuminated with visible laser (532 nm) and IR laser (1064 nm). In these nanowire devices, we have observed faster rise and decay times which depend on the laser power and the applied electrical bias voltage. The best photoresponsivity measured among the nanowire devices is in the order of ~300 A/W which is ~10000 times larger than the ultrathin nanosheets of Bi_2_Se_3_^21^.

Figure 1a shows the field emission scanning electron microscopy (FESEM) image of the deposited Bi_2_Se_3_ flake where surface looks clean and smooth. Note that a thin flake of interest was first localized under FESEM but was never exposed to Ga ion imaging. Green colour rectangle shows the area selected for the milling and the small square was used for the alignment of electron beam and Ga ion beam. FIB milling was performed for the fabrication of nanowire geometry by etching out selected portion of the flake as shown in the Fig. 1b. Typical length and width of the fabricated nanowire were in the range of 10–11 μm and 180–220 nm, respectively. The metal electrodes of Pt were deposited on the fabricated nanowire by using FIB based gas injection system (Zeiss Auriga). Figure 1c displays the FESEM image of FIB fabricated Bi_2_Se_3_ nanowire device used for the photocurrent measurement in this study. The linear current voltage relationship shown by the black (light off) and the red (light on) curves in the inset of Fig. 1c indicates good ohmic contact of the electrode with the nanowire and change in photoconductivity is consistent with the previous IV measurements on nanostructured materials^23^,^24^. A three dimensional schematic representation of the nanowire device along with the experimental scheme for photocurrent characterization under the light illumination is presented in Fig. 1d. The crystalline quality of the Bi_2_Se_3_ flakes was observed under high resolution transmission electron microscope (HRTEM) (Fig. 2) which is described in the method section.

The Bi_2_Se_3_ nanowire devices were characterized for their time dependent photoresponse properties to broad spectral laser excitations using visible laser (532 nm, ~32 mW) and infrared laser (1064 nm, ~29 mW). Prior to any photocurrent measurements, laser illumination was guided perpendicularly over wide area covering the entire device as shown schematically in Fig. 1d and was adjusted for maximum photocurrent signal. The estimated max illumination intensities for IR laser and for visible laser were ~29 mW/cm^2^ and ~32 mW/cm^2^, respectively. A Keithley 2634B source measure unit (SMU) was used for the electrical characterization of the fabricated photodetector devices.

First, we have characterized the photocurrent dynamics of the nanowire photodetector for cyclic exposures of visible light (laser power ~32 mW, 532 nm) to study the stability and repeatability of the photocurrent as shown in the Fig. 3a. A constant bias voltage was applied between source and drain terminals to measure the time dependent current changes in the device under the dark and illuminated conditions. Photocurrent (*I*_*Ph*_) was extracted by subtracting the dark current (*I*_*dark*_) from the measured current in presence of laser light (*I*_*ligh*t_) i.e. *I*_*ph*_ = *I*_*light*_*-I*_*dark*_^24^,^25^,^26^. The measured photocurrent with a bias voltage of 75 mV is observed to increase rapidly once the device is illuminated (ON) with 532 nm visible laser. The photocurrent drops dramatically with removal of the light (OFF). The colours in Fig. 3a indicate the cycles of laser light in its ON and OFF states. The corresponding sequential increase and decrease in photocurrent for ON and OFF states, respectively, have been observed for many repeated cycles and found to be reproducible as shown in Fig. 3a. The fast photoconductivity detection dynamics between OFF and ON cycles of light exposures demonstrate the suitability of the fabricated Bi_2_Se_3_ nanowire devices as photo switches or high quality photodetectors.

The response and decay times are important parameters for any photodetector. We have used following exponential equations to calculate the response and decay times, i.e., 
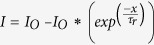
 and 
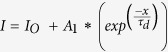
respectively.

Figure 3b represents the data for one ON/OFF cycle taken from Fig. 3a and it shows that photocurrent increases rapidly to ~7 nA once the light is on. The measured time dependent photocurrents under the ON and OFF state are fitted with above exponential rise and decay equations yielding time constants of 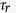
~ 520 ms and 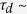 730 ms, respectively. The fast increase and decrease in photocurrents under ON and OFF states, respectively, also attribute to the fact of less density of trapped electronic states induced by defects. There are numerous reports mentioning improvements in rise or decay time constants when measured at nanowire or ribbon scale. Time dependent dynamics of photocurrent rise have been investigated with varying applied bias as shown in Fig. 3c. We observe that photocurrent can be noticed significantly if the applied bias voltage is more. For example, photocurrent is increased to ~15 nA when the applied bias is 125 mV. The response and decay times have been measured for every voltage dependent photocurrent rise and decay curves and the result is plotted in Fig. 3d. The faster response time, 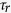 ~370 ms and decay time, 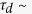
625 ms are observed for the bias voltage 125 mV under the illumination of 532 nm visible laser with power density 32 mW/cm^2^. These values of 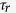
and 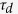 represent much faster response for our Bi_2_Se_3_ nanowire based photodetectors compared to photodetectors made from other novel layered materials like MoS_2_ (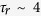 sec and 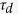 ~ 9 sec)^24^. The bias voltage dependent increment of photocurrent was reported previously and it has been attributed to the increased drift velocity and reduced transit time for the carriers at high bias, as *T*_*t*_ = *l*^*2*^/*μV*_*ds*_, where *l* is the device length, *μ* is the carrier mobility and *V*_*ds*_is the bias voltage^24^. This indicates further improvement in response and decay times can be achieved with the application of higher bias voltages and Bi_2_Se_3_ nanowire photodetector can be suitable for the fast varying optical signal.

Evolution of photocurrent is further characterized under the illumination of IR light source (1064 nm) for various applied bias voltages as shown in Fig. 4. Bi_2_Se_3_ nanowire device has been exposed for the repetitive ON/OFF cycles of IR light with power density ~29 mW/cm^2^ and significant rise/decay in photocurrent (~10 nA, Fig. 4c) was observed (Fig. 4a). Data in Fig. 4b shows the kinetics of photocurrent rise/decay due to single exposure of the laser light ON/OFF cycle, respectively. Exponential rise or decay equations as shown above are used to study the dynamics of 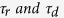. The effect of applied bias voltages on the dynamics of photocurrent rise/decay is studied while keeping IR laser light intensity constant (~29 mW/cm^2^) and is shown in Fig. 4c. The 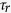 (950 ms) and 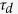 (900 ms) are measured for bias voltage 25 mV, these values have been improved further to 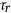 (550 ms) and 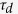 (400 ms) with a bias voltage of 150 mV. The dependency of time constants on the applied bias voltage has been plotted in Fig. 4d.

Photocurrent detection in nanowire device due to IR light has been further characterized to check dependency of the photocurrent on the incident power density of the laser while keeping the bias at constant voltage. Photocurrent of about ~2 nA is observed for 5 mW/cm^2^ but small fluctuation in current is noticed at the photocurrent saturation plateau when the bias voltage is 150 mV. For power densities <5 mW/cm^2^, no noticeable measurement in photocurrent was found, above this threshold power, significant photocurrent rise is detected which is shown in Fig. 5a. Further, the power law dependency has been studied and shown in the Fig. 5b. We have used a simple power law relation, *I*_*ph*_ ~ *P*^*Θ*^, to fit the data of photocurrent measured at different powers. Consequently, a sub-linear increase is found in the photocurrent with increasing power density for the incident illumination systematically. This increment in linearity is the characteristic feature of a photocurrent indicating minimal contribution from the thermoelectric current as Bi_2_Se_3_ is a very good thermoelectric material. Light sensitive, polarization dependent generation and control of photocurrents originating from topological surface states in Bi_2_Se_3_ have been studied already^20^. Fit with exponent Θ ~ 0.66 shows the photocurrent response to laser power density at 1064 nm wavelength (the red curve Fig. 5b).

Further, photoresponsivity of the photodetector is measured using the relation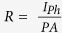 where, *R* = *photoresponsivity (A*/*W), I*_*ph*_ = *photocurrent, P* = *Light intensity* and *A* = *effective area of the nanowire.* We have calculated the photoresponsivity for both visible and near infrared wavelengths. The photoresponsivity of Bi_2_Se_3_ nanowire as a function of laser power density is plotted in Fig. 5b and we have found a value of R ~ 300 A/W for bias voltage 150 mV with laser power 5 mW/cm^2^. This photosensitivity is a huge improvement (~10,000×) compared to the previous photoresponse reports on ultrathin Bi_2_Se_3_ nanosheets^21^. The increase in light intensity decreases the photoresponsivity which suggests that light absorption efficiency increases or reaches to a maximum value in nanowire devices. From these values of responsivities, we have estimated bias voltage dependent detectivity (*D)* and external quantum efficiencies (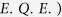
27,28,29 as, 
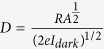
 and 
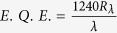
. The voltage and power dependency of these values have been plotted separately and can be found in the supplementary information. At constant bias of 150 mV and laser power density 5 mW/cm^2^, the detectivity and E.Q.E. are found ~7.5*10^9^ Jones and 3.4*10^4^% respectively. The detectivity of the device is shown by a blue curve Supplementary information and the decrease in detectivity is observed for increase in power density of the laser light indicating the device performs better for low laser power. Since our estimated values of EQE and detectivity are bias dependent more reasonable EQE values can be calculated through ultrafast transient absorption measurements^30^.

Photoconductivity at visible and NIR wavelengths can be explained as efficient light absorption in FIB fabricated Bi_2_Se_3_ nanowire device and generation of more electron hole pairs with the help of a bias voltage. The present photodetectors based on topological insulator materials and their device performance parameters have been compared with our results and summarized in Table 1. It is supported that photodetectors made from nanowires show enhanced optoelectronic properties as compared to their bulk counterparts^31^,^32^. To check the width of nanowire and its dependency on photoresponsivity we have performed photoconductivity experiments on larger width and have found a decrease in the photoresponsivity (Supplementary Fig. 3). Further to check the enhancement in responsivity, we have carried out photoconductivity experiments before and after milling of pristine flakes (micron size sheets). The results along with the details of the samples before and after FIB milling are appended in the supplementary information (Supplementary Figure 4a & b). The quantum confinement effects taking place may be due to fabrication of a Bi_2_Se_3_ nanowire from ultrathin flake results into a large surface-to-volume ratio and may give rise to a new electronic state. Being a layered material Bi_2_Se_3_ consists of both electronic and mechanical confined effects, correspondingly, the changes in photo-absoprtion spectra as a function of size and thickness of the flake have been observed earlier^12^,^19^. The theoretical work on topological insulators and Dirac semimetals reported interesting quantum confinement effects such as bulk band gap opening at the Dirac points, strong anisotropic effects along different directions, spin splitting for the surface states (Rashba)^4^. Cylindrical nanowires of Bi_2_Se_3_ were studied theoretically for their persistent charge and spin currents^33^. The four orders of change in the photoresponsivity reported here for FIB fabricated nanowires could be due to (i) quantum confinement effects in the nanowire geometry, (ii) highly efficient carrier concentration,(iii) good ohmic contacts, (iv) less defective material and combined with the surface electronic properties of topological insulators. Thus there is always possibility of gallium contamination during FIB milling and fabricated nanowire should have some defect states which is interesting for future studies since there is strong interest in investigating topological surface states, defects and their robustness^34^,^35^. The slow photoresponse observed as considering the fact that the topological states are only protected for a time which is shorter than a picosecond and we have observed its bulk contributions with gallium impurities.

Bi_2_Se_3_ possesses special electronic properties that surface states form a single Dirac cone inside a large bulk band gap and it is theoretically and experimentally investigated as 3D TIs material^17^. The origin of photocurrent in such topological insulators is a complex phenomenon and explained as interplay between orbital and the Zeeman coupling of the light to the surface electrons^36^. It is also shown that the contributions from helicity-independent photocurrent dominates over the helicity-dependent photocurrent which is found very small when circularly polarized light was incident obliquely^36^. We assume the fact that most TIs made to date have not been completely insulating in the bulk and due to impurities it could be conducting. Thus overall photocurrent observed here could be the combination of bulk contributions, photo galvanic, photon drag^20^ or thermoelectric effects. The results presented here suggest more theoretical and experimental work needed in understanding the origin of photocurrent enhancements in Bi_2_Se_3_ nanowire photodetector which has potentiality in analogy with graphene based photodetectors.

In conclusion, the photocurrent dynamics of Bi_2_Se_3_ nanowires fabricated from the flake is studied here. We demonstrate successful fabrication of Bi_2_Se_3_ nanowires by FIB milling method using the flake as a starting material. Fabrication of nanowire reported here is a very simple and straightforward method because Bi_2_Se_3_ is a layered material and few layers (atomically thin sheets) of it can be exfoliated on the substrate for FIB milling by a simple scotch tape (micromechanical cleavage) method. The fabricated Bi_2_Se_3_ nanowire device has shown broad spectral response (visible and NIR). The response, rise and decay time, of FIB fabricated Bi_2_Se_3_ nanowire device is very fast and excellent photocurrent stability and repeatability have been noticed. The photocurrent showed dependency on the laser power density and applied bias voltages. The high photoresponsivity is observed which is ~4 orders of magnitude improvement as compared to ultrathin Bi_2_Se_3_ nanosheets^21^ and could be due to the quantum confinement effects, reduction of good crystalline quality flakes into nanowire/nanostructures and large surface to volume ratio. Our work suggests the feasibility of designing nanowires of topological insulators from the high quality deposited flakes of the same material using FIB milling technique and opens pathway for integrating TIs into nano optoelectronic applications. The thin nanowires of TIs could be exploited further to study very exotic low temp electronic properties or topological surface state properties of topological insulators. Moreover ultrasensitive TIs based nanowire photodetector is demonstrated here which can be explored further for various possible applications in optical communications, imaging, thermal detection, spectroscopy, remote sensing etc.

## Methods

### Device Fabrication

**Figure 1:**
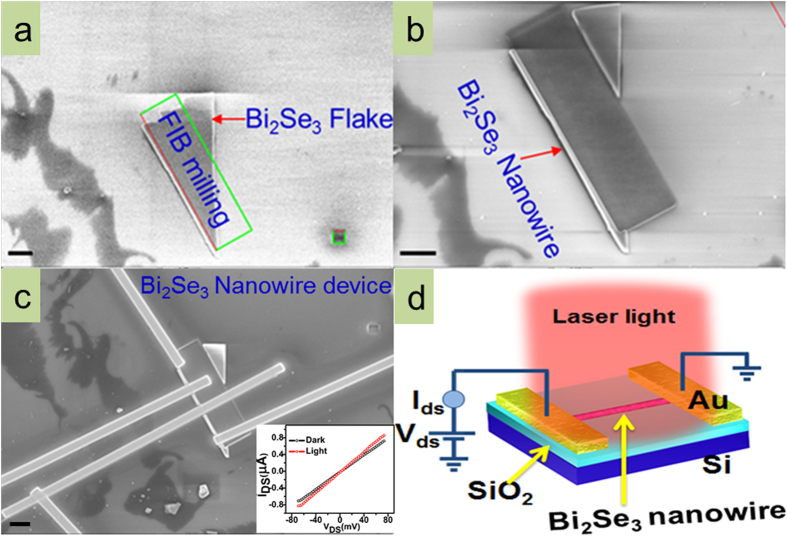
Focused ion beam fabrication and photo detection in Bi_2_Se_3_ nanowire. **(a)** Deposition of Bi_2_Se_3_ flake on SiO_2_/Si substrate by using scotch tape method. Rectangle indicates the area of flake selected to etch by Ga^+^ ion milling. Small square indicates the area used to align electron and ion beam at the same location. **(b)** Selective portion of the flake left out after FIB milling resembles into a nanowire. **(c)** Metal electrodes (Pt) deposited on the Bi_2_Se_3_ nanowire for optoelectrical characterization. Inset represents current-voltage relationship of the device under dark conditions (black curve) and when the device was illuminated under light (red curve). **(d)** The 3D schematics of the Bi_2_Se_3_ nanowire device illustrating optoelectrical measurement setup and laser light illumination. Scale bar is 1 μm.

Devices of Bi_2_Se_3_ nanowires were made by using scotch tape method and focused ion beam (FIB) microscopy. Prior to deposition of flakes on SiO_2_/Si, substrates were cleaned with acetone, isopropanol, methanol and treated with oxygen plasma for ~5 min. Bi_2_Se_3_ ( 99.999% CAS#12068-69-8) material was procured from company Alfa Aesar. By using micromechanical cleavage (scotch tape method), thin flakes of Bi_2_Se_3_ were exfoliated on SiO_2_/Si substrates having predefined gold pads. This method yields random sizes and thicknesses of the Bi_2_Se_3_ flakes which were observed under optical (Olympus) and electron microscope (Zeiss Auriga, Fig. 1a). Once thin flakes were localized, nanowires of Bi_2_Se_3_ (Fig. 1b) were fabricated by FIB milling process (Ga+ ions) and thin flakes deposited on SiO_2_/Si substrates were used as a starting material. Metal electrodes of platinum or tungsten were deposited on the nanowire by using FIB based gas injection system (Zeiss Auriga). The devices fabricated were loaded in the probe station setup (Cascade Microtech EPS150TRIAX) which has shield enclosure (EPS-ACC-SE750) for low signal measurements.

### TEM and HRTEM characterization

**Figure 2:**
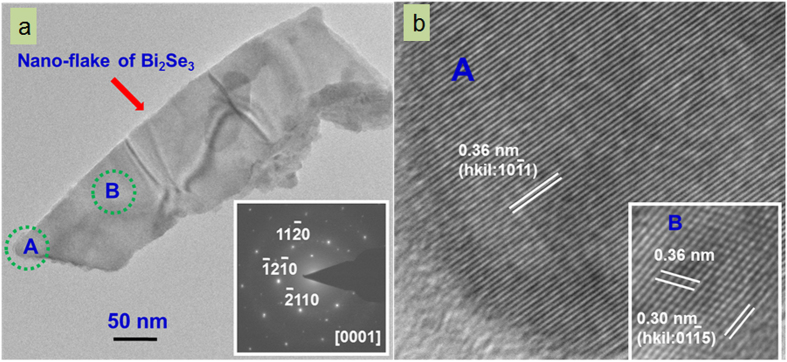
TEM characterization of thin Bi_2_Se_3_ flake. **(a)** TEM image of the flake first dispersed in isopropanol and deposited on TEM grid. Inset represents the SAED pattern acquired from the same flake. **(b)** HRTEM image of location A in fig (**a**) and inset is the HRTEM image of location B in fig (**a**).

High resolution transmission electron microscopy (HRTEM model: Tecnai G2 F30 STWIN assisted with field emission gun and with an electron accelerating voltage of 300 kV) was employed on Bi_2_Se_3_ samples to reveal microstructural information even up to atomic scale including the reciprocal space. In general, a thin flake-type morphology with a facetted-edge was observed in the microstructure. As an illustrative example, Fig. 2a depicts a flake of dimensions about 100 nm in breadth and 3500 nm in length. Although the thickness of these flakes was not measured in the present investigations, however, the specimen being a good electron beam transparent, the consequent thickness may always be presumed approximately between 10 to 20 nm. A corresponding selected area electron diffraction pattern (SAEDP), inset in Fig. 2a, recorded from the flake microstructure in reciprocal space exhibited a crystalline pattern aligned along [0001] zone axis showing a hexagonal symmetry of a rhombohedral phase of Bi_2_Se_3_ (space group: R
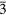
m, lattice constants: a = b = 0.414 nm, c = 2.863 nm, ref.: JCPDS card no. 33-0214). A set of important planes of the rhombohedral crystal structure of: 11
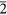
0, 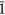
2
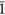
0, 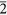
110with an interplanar spacing of 0.21 nm, are marked on the SAEDP (inset in Fig. 2a). The atomic scale images of an edge and inside regions of the flake (regions marked as A and B in Fig. 2a) are displayed as in Fig. 2b (atomic scale image of A) and an inset of Fig. 2b (atomic scale image of B). A stacking of 10
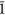
1 planes revealing a crystalline structure of flake morphology in real space is clearly visible from the micrograph (Fig. 2b). The inset in Fig. 2b (magnified view of region B in Fig. 2a) further demonstrates the crossover atomic planes of Bi_2_Se_3_ of 10
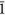
1 and 01
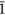
5 with the corresponding interplanar spacings of 0.36 and 0.30 nm, respectively.

## Additional Information

**How to cite this article**: Sharma, A. *et al.* High performance broadband photodetector using fabricated nanowires of bismuth selenide. *Sci. Rep.* **6**, 19138; doi: 10.1038/srep19138 (2016).

## Supplementary Material

Supplementary Information

## Figures and Tables

**Figure 3:**
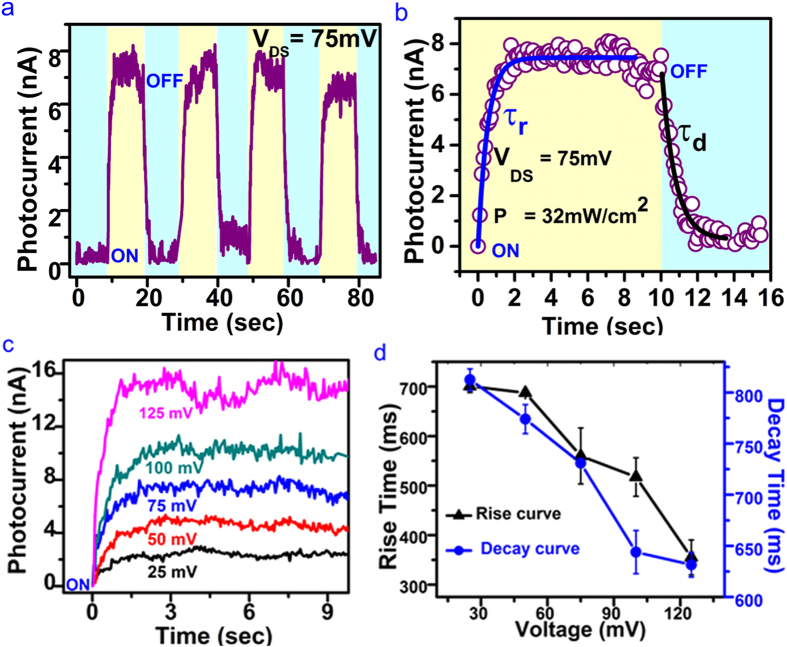
Visible light (532 nm) induced photocurrent generation in Bi_2_Se_3_ nanowire. **(a)** The data show the measurement of photocurrent (*I*_*ph*_) under a constant bias voltage V = 75 mV and laser power density ~32 mW/cm^2^. Photocurrent was extracted from the measurements done in absence of light (*I*_*dark*_) and in presence of light (*I*_*light*_) i.e. *I*_*ph*_ = *I*_*light*_ − *I*_*dark*_. ON (yellow colour) indicate the duration for which light was turned on and OFF (sky blue) is the duration when light was turned off. **(b)** Indicate the selection of the data of (**a**) used to calculate the response time 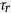 (fit curve is shown by the blue curve) and decay time constant 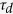
(fit curve is shown by the black curve). **(c)** Show the bias voltage dependent characterization of photocurrents. For the clarity, if the measurements done on different time scale of the light on/off cycles were synchronized to zero. Different colour curves indicate the photocurrent rise with the increase in applied voltage (Δ25 mV). **(d)** The black triangles and the blue dots curves represent the rise and decay time measured as a function of applied bias voltage respectively. Error bar is the standard deviation performed on different data points of the measurements.

**Figure 4:**
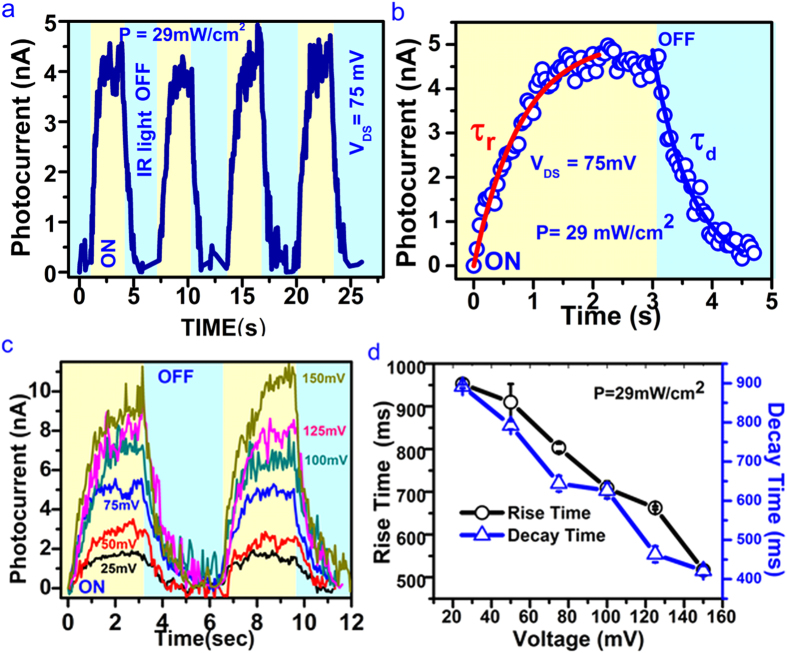
NIR (1064 nm) induced photocurrent generation in Bi_2_Se_3_ nanowire. **(a)** The blue curve represents the photocurrent (*I*_*ph*_) measurements done for different ON/OFF cycles of IR laser light at constant bias voltage V = 75 mV and laser power density ~29 mW/cm^2^. Yellow and sky blue colours indicate time duration when light was ON and OFF respectively. **(b)** Show the calculation of response (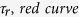) and decay (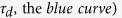
time constants from the data shown in **(a)**. **(c)** Shows the photocurrent measurements done for different bias voltages. The convincing increase in photocurrent is visible as a function of increase in applied bias voltage. The colour of applied bias voltage represents the data of the curve in same colour. **(d)** The black circles and the blue triangle curves represent the rise and decay time measured as a function of applied bias voltage respectively. Error bar was calculated from the standard deviation.

**Figure 5:**
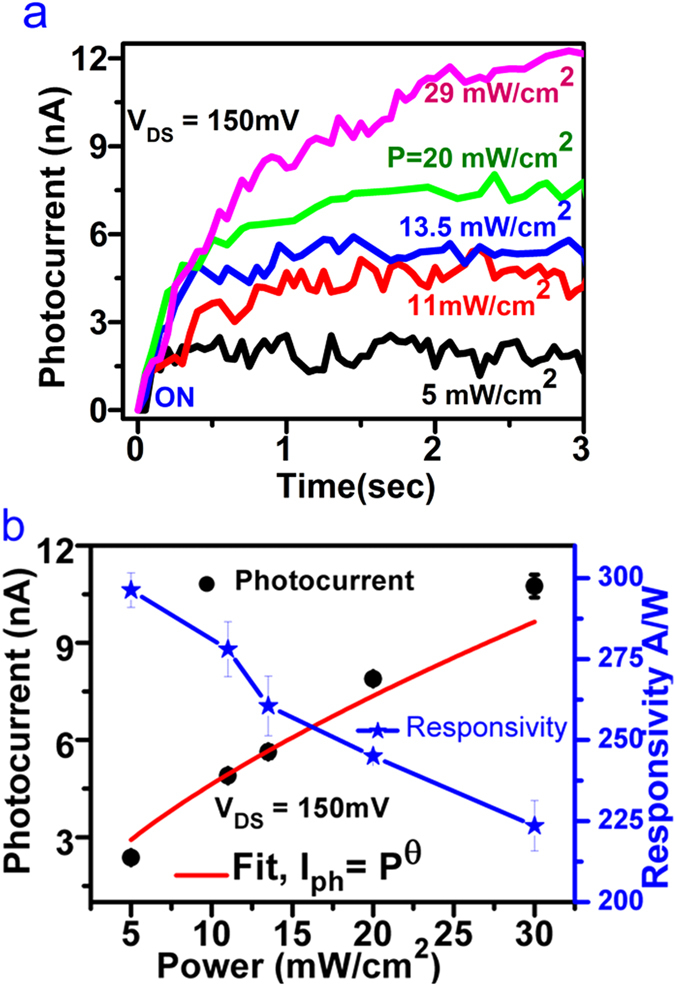
Laser power dependent dynamics of the photocurrent. **(a)** The rise in the photocurrent was monitored as a function of the laser power and bias voltage of 150 mV was kept constant. Noticeable photocurrent was measured when the laser power density was 5 mW/cm^2^ (black curve). The different curves red, blue, green and magenta were measured at power densities of 11, 13.5, 20 and 29 mW/cm^2^ respectively. ON indicates that laser light was made on. **(b)** Shows the characteristics of photocurrent as a function laser power (black dots) fitted for the power law relation *I*_*ph*_ = *P*^*Θ*^ (red curve). Decrease in the photoresponsivity as a function of increase in laser power was observed (blue curve).

**Table 1:** Topological insulator based photodetectors.

Material	λ (nm)	R (AW^−1^)	I_light_/I_dark_	G	D (Jones)	Rise Time (s)	Decay Time (s)	Ref.
Bi_2_Se_3_ nanowire	1064	300	1.0013	~350	7.5 × 10^9^	0.55	0.4	Our work
Sb_2_Te_3_ film	980	21.7	2.36	27.4	1.22 × 0^11^	238.7	203.5	13
Polycrystalline Bi_2_Te_3_	1064	3.03 × 10^−5^	1.0004	3.85 × 10^−5^	—	—	—	14
Graphene- Bi_2_Te_3_	980	10	—	11	—	—	—	15
Bi_2_Se_3_ nanosheet (exfoliated)	—	20.48 × 10^−3^	4.3333	—	—	0.7	1.48	21
