# Supplementary material for: High performance broadband photodetector using fabricated nanowires of bismuth selenide
Source: Sci Rep. 2016 Jan 11;6:19138. doi: 10.1038/srep19138 (PMC4707481; doi:10.1038/srep19138)
Supplement: Supplementary Information [file srep19138-s1.pdf]

High performance broadband photodetector using fabricated nanowires of bismuth selenide

*Alka Sharma[1,2], Biplab Bhattacharyya[1,2], A. K. Srivastava[1,2], T. D. Senguttuvan[1,2] and Sudhir Husale[1,2]**

[1]Academy of Scientific and Innovative Research (AcSIR), National Physical Laboratory, Council of Scientific and Industrial Research, Dr. K. S Krishnan Marg, New Delhi-110012, India.

[2] National Physical Laboratory, Council of Scientific and Industrial Research, Dr. K. S Krishnan Marg, New Delhi-110012, India.

**e-mail: husalesc@nplindia.org*

Supplementary Information contents

1. **Bias voltage dependent responsivity, detectivity and external quantum efficiency**
2. **Formulae's used in the study**
3. **Control Experiments**
4. **Width dependence photoconductivity**
5. **Control experiment to show the enhancement in photoconductivity before and after milling of Bi_2Se_3 flake**
6. **Photoconductivity measurement done on FIB fabricated nanowire device no.3**

1. Bias voltage dependent photoresponsivity, detectivity and external quantum efficiency

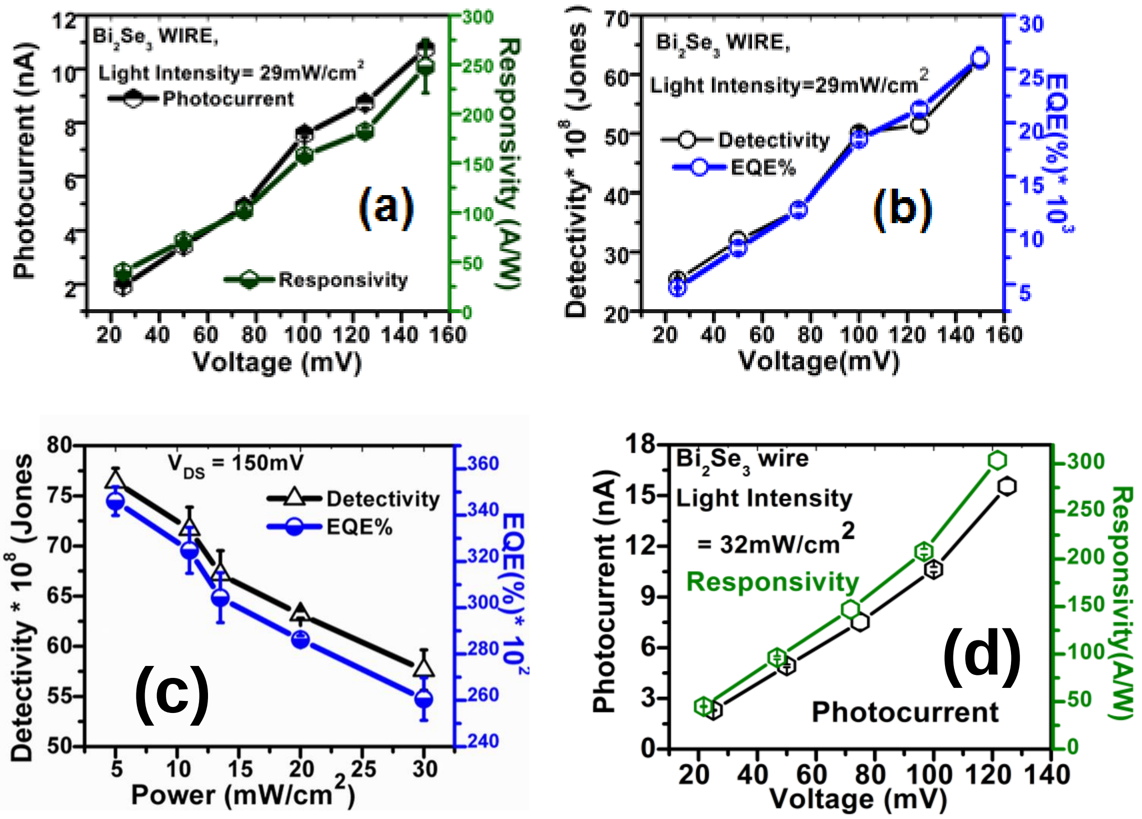

Supplementary Figure 1 | Bias voltage dependent characterization of photoresponse under the illumination of IR laser: **(a)** Photocurrent (black curve) and responsivity (green curve) plotted as a function of applied bias. **(b)** The bias voltage dependency of the detectivity (black curve) and external quantum efficiency (blue curve). **(c)** Detectivity (black curve) and external quantum efficiency (EQE, blue curve) were plotted as a function of laser power density. As expected, higher detectivity or EQE was observed at lower power density. Measurements were repeated to get the error bar (standard deviation). (d) The bias voltage dependent responsivity and photocurrent measurements done when the device was illuminated 532 nm laser.

2. Formulae's used in the study

Photocurrent

$$I_{ph} = I_{light} - I_{dark}$$

Photoresponsivity

$$\mathrm{R} = \frac{I_{ph}}{P*A}$$

Detectivity $$D = \frac{R\sqrt{A}}{\sqrt{2eI_{dark}}}$$

External Quantum Efficiency $$E.Q.E = \frac{hcR_{\lambda}}{e\lambda}$$

Rise Equation $$I = I_o - I_o * (e^{\frac{-x}{\tau r}})$$

Decay Equation $$I = I_o + A_1 * (e^{\frac{-x}{\tau_d}})$$

Where $I = \text{current(nA)}$, $I_{Ph} = \text{Photocurrent}$ (nA)

R = Responsivity (A/W), P = Light Intensity (mW/cm^2)

A = Effective area of the wire (cm^2),

e = electronic charge (C)

D = Detectivity (Jones),

E.Q.E.= External Quantum Efficiency

λ = Wavelength of light (nm)

$\tau_r = Rise\ Time$ (ms) , $\tau_d = Decay\ Time\ (ms)$

I_O = Initial value of the current, A_1 = Independent Variable

3. Control Experiments

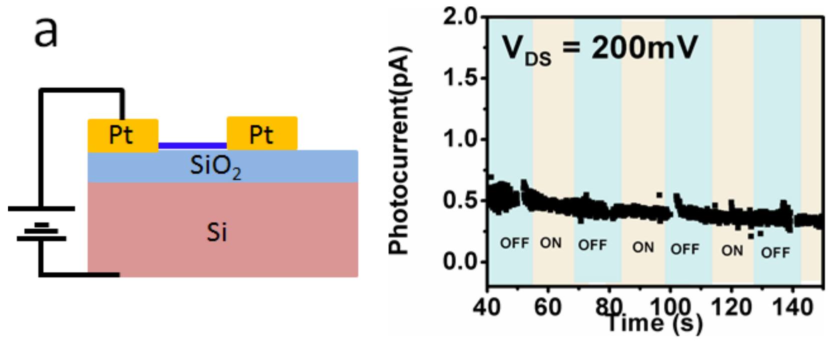

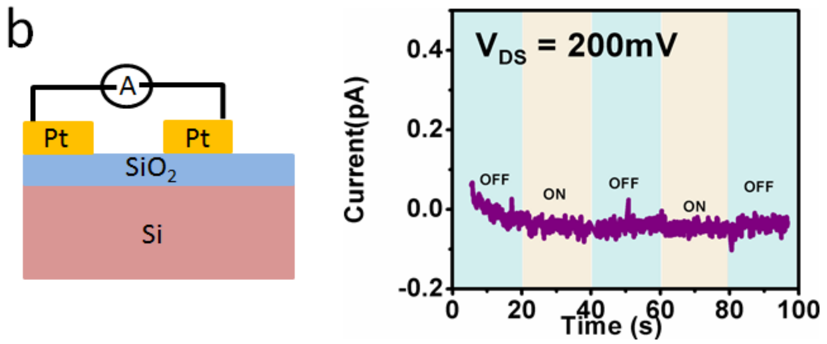

Supplementary Figure 2a&b Fig a shows the schematic of the leak current measurements and experimental data is shown on the right side for the same. Fig b shows sio2 film characterized after gallium milling for 20 sec, area of illumination (25 x25 μm^2) and current was ~50 pA

4. Width dependence photoconductivity

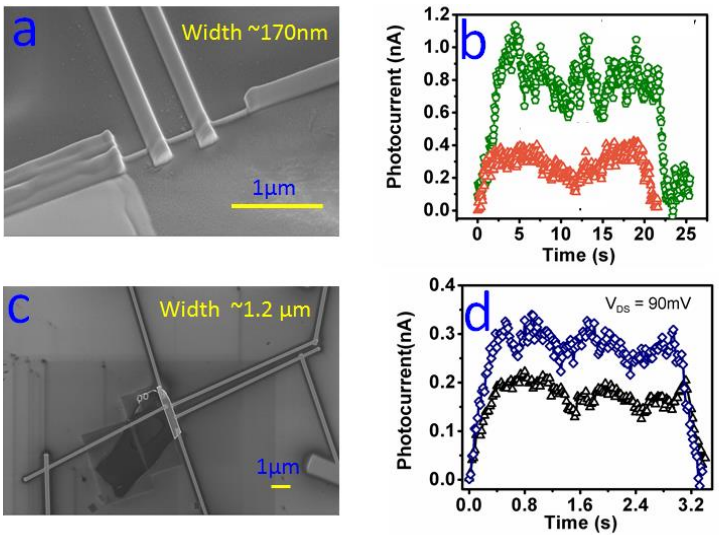

Supplementary Figure 3 a-d show the fabricated samples used for width dependence study. Photocurrent data for ~170nm is shown in fig b whereas photocurrent data for width 1.2um

is shown in fig d. Photoconductivity measurements were carried out between different electrodes (2probe measurements). About 3x enhancement in photocurrent was observed for nanowire sample.

5. Control experiment to show the enhancement in photoconductivity before and after milling of Bi_2Se_3 flake

We have carried out photoconductivity experiments on pristine flakes (micron size sheets) and some portion of the same flake was milled out by FIB to get the nanowire (width ~ 270 nm) which is shown in the following figure. The photoconductivity measurement data clearly shows the enhancement in the photoresponsivity (~52 A/W for fig a and ~240 A/W for fig b). In both the cases, the illumination of laser light was global illuminating the whole device, with spot diameter was ~ 1mm^2, wavelength 532 nm and bias voltage 500 mV.

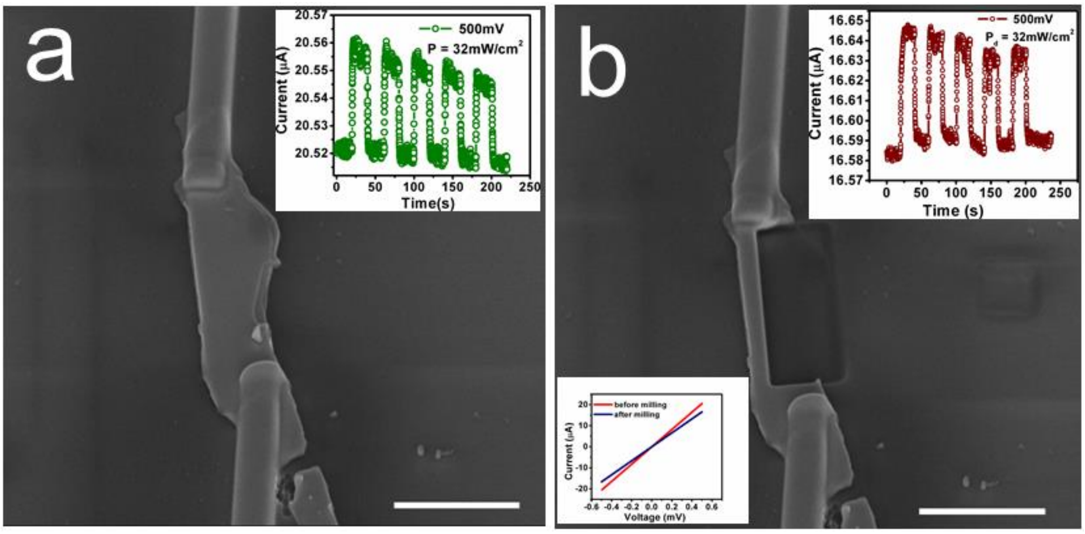

Supplementary Figure 4 a&b are the FESEM images of Bi_2Se_3 flake before milling (fig a) and after milling (fig b). Inset in fig (a) represent the photoconductivity measurements done on the pristine flake at constant bias voltage. The increase in current is due to the illumination of laser light on the device and decrease in current means the laser light was switched off. The inset in the top fig (b) exhibits the photoconductive measurements at the same constant bias and down inset represents the dark current measurements done before milling (red curve) and after milling (blue curve). Scale bar is 2 μm.

6. **Photoconductivity measurement done on FIB fabricated nanowires to check the reproducibility for device no. 3**

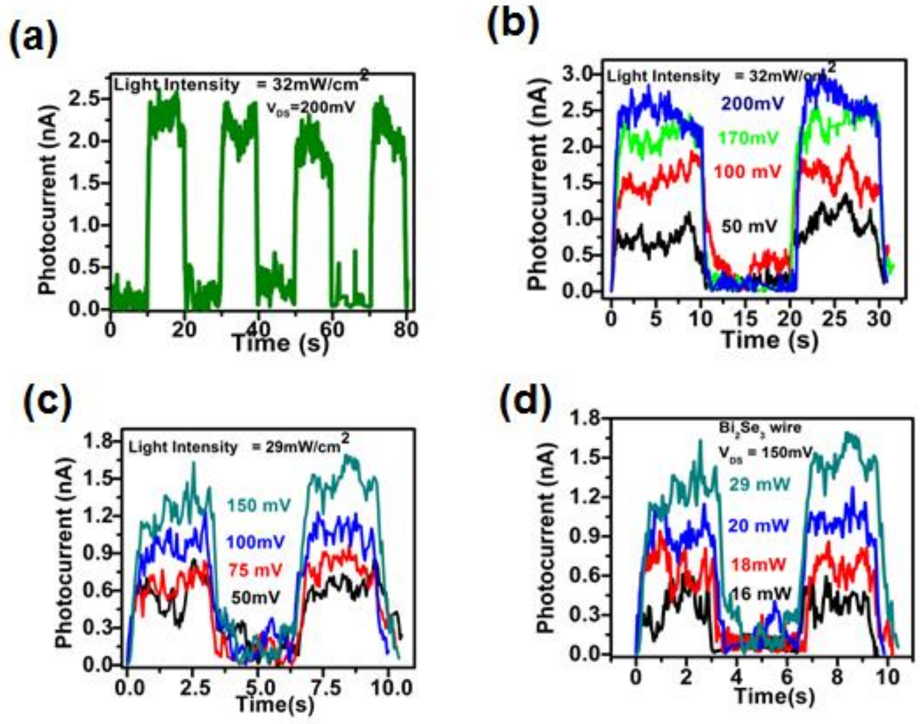

Supplementary Figure 5 | Characterization of time dependent photoresponse under the illumination of visible (a&b) and IR laser (c&d): (a) Cyclic evolution of photocurrent (I_{ph}) with a constant bias voltage V = 200 mV for visible laser light (532 nm) ON and OFF states with laser power density ~ 32 mW/cm^2. Photocurrent is extracted from the measurements done in absence of light (I_{dark}) and in presence of light (I_{light}) using the relation, $I_{ph}=I_{light}-I_{dark}$. **(b)** The bias voltage dependent generation of the photocurrent under visible light illumination. **(c)&(d)** The time dependent photocurrent response for different bias voltages and power density of the infrared laser light (1064 nm) respectively.
